# HRPC: A need for multidisciplinary approach

**DOI:** 10.4103/0970-1591.30252

**Published:** 2007

**Authors:** Nitin S. Kekre

**Affiliations:** Editor in Chief, IJU, Professor, Department of Urology, Christian Medical College, Vellore - 632 004, Tamil Nadu, India. E-mail: editor@indianjurol.com

Hormone refractory prostate cancer remains a very challenging problem for both urologists and oncologists. Significant development has occurred in this area and the role of multi-disciplinary treatment is increasing. Urologists needs to work in collaboration with medical oncologists, palliative care consultants, specialized nurses, and social workers to improve quality of life. Dr. Makarand Kochikar and his co-authors have provided a very exhaustive review of this topic, and it will be a good reference for practicing urologists. I sincerely thank them for their effort.

We are all very proud of our ancient heritage and culture. However, being groomed under the umbrella of western medicine, we are ignorant about the medical history of ancient India. The names of Charak and Sushruta are very well known to us, but we do not know their urological contributions. In this issue, Dr. Sakti Das takes us through the scene of urology in ancient India. I am gratefully indebted to Dr. Sakti Das for his contribution.

Vasectomy is a very simple operation for all of us. It would be very difficult for anybody to believe that this simple snip can be a cause for lawsuits of medical negligence. There are several issues regarding vasectomy follow-ups, which are not carefully adhered to. Many times the excised was not sent for histopathological confirmation. Two consecutive negative semen samples may not be obtained before declaring the person′s sterile. Drs. Dhar and Jones provide an in-depth review on this topic should provide a food for thought.

Though last year was challenging, it was a fruitful year for the journal. The beginning of the New Year provides the opportunity to audit the performance of the last year and plan for the future. Last year we decided to publish IJU quarterly and to bring out the issues on time, and I am happy that we managed to achieve this target of publishing quarterly issues on time. All the submissions are subjected to formal peer review. As the entire process of manuscript submission, peer review, and proofreading is being done online, it has completely eliminated the problems of manuscript loss, postal delay, and has shortened the publication time. The approximate time from the submission to the publication of an article is 120 days. We have been able to print colored photographs without any financial burden to the authors. The process of peer review is completely blinded, which maintains impartiality and avoids personal biases. Evidently, this has improved the quality of the papers published in the journal. The journal website is functioning well. The free online access policy has increased the readership of the journal and it is being accessed widely. There were more than 1,30,000 visitors to the IJU website last year.

The format of the journal is now fairly standardized. Each issue has a combination of review articles on current urological topics by well-known national and international experts original articles and symposia on important clinical problems. We have reduced the number of case reports to approximately 5-6 in each issue. The uroscan provides a good review of the current published urological literature, while the evidence-based urology corner attempts to provide a balanced scientific, but critical commentary on controversial urological issues. It's my sincere hope that you would have found these articles interesting and beneficial. I would be keen to have your feedback in this regard, which will help us to plan for the future.

Let me once again wish you all a very happy 2007.

